# Morphometric Analysis of the Proximal Femur With Its Clinical Correlation in Eastern Uttar Pradesh Region

**DOI:** 10.7759/cureus.28780

**Published:** 2022-09-04

**Authors:** Mayank Gupta, Deepa Devadas, Chetan Sahni, Amit Nayak, Praveen K Tiwari, Anand Mishra

**Affiliations:** 1 Forensic Medicine, Institute of Medical Sciences, Banaras Hindu University (BHU), Varanasi, IND; 2 Anatomy, Institute of Medical Sciences, Banaras Hindu University (BHU), Varanasi, IND

**Keywords:** femur length, fovea capitis, femur head diameter, neck shaft angle, morphometry, proximal femur

## Abstract

The anthropometry of the proximal femur holds great clinical significance in designing implants and prostheses for proximal femoral fractures and hip joint arthroplasties. Surgical fixation with a properly matched prosthesis plays a crucial role in improving long-term treatment outcomes and preventing post-operative complications such as osteolysis with aseptic loosening and increased load. The femur is also one of the most commonest used bones for stature estimation. Often during forensic investigations, only fragmented remains of femur are found available from which femoral length is estimated by application of linear regression equations. The estimated femoral length thus obtained is used for stature estimation of the unidentified individual. This study has measured nine bony parameters from the proximal femur in a total of 96 dry femora. These measurements include the vertical head diameter, neck diameter, neck thickness, neck length, neck shaft angle, the transverse diameter of the fovea, longitudinal diameter of the fovea, foveal depth, and the intertrochanteric line length. In addition, the total length of the femur was also measured. The results were tabulated and statistically analyzed using SPSS software, version 25. The mean femoral head diameter was observed to be 41.59±3.25 mm, mean foveal depth was found to be 2.95±0.75 mm, mean foveal transverse and longitudinal diameters were observed to be 11.38±2.35 mm and 15.94±3.37 mm, respectively. The mean neck diameter was 29.45±3.33 mm. Mean neck length and neck thickness were observed to be 36.06±4.94 mm and 27.61±2.71 mm, respectively. Neck shaft angle was noted to range from 109° to 128°, with a mean of 119.08°±5.18°. The mean length of the inter-trochanteric line was measured to be 41.92±3.9 mm. The mean femoral length was observed to be 42.11±2.91 cm. Significant positive correlations were found between the various measured proximal morphometric parameters of the femur. The length of the femur showed a maximum positive correlation with the vertical head diameter, followed by the neck diameter, thickness, and foveal depth. The findings of this study can throw further light on the existing data. They can serve as a guideline for designing better-matched prostheses and implants for hip surgeries in the eastern Uttar Pradesh population.

## Introduction

The femur, the longest and strongest bone in the body [1], holds great clinical significance in the world of anatomists, forensic experts, orthopedic surgeons, and sports physicians. The length of the femur is associated with a striding gait and its strength with the weight and muscular forces it is required to withstand [1].

The femur has a proximal end, shaft, and distal end. The proximal end consists of the head, neck, and greater and lesser trochanters. The spheroidal head of the femur articulates with the acetabulum of the hip bone to form the hip joint and lies within the joint capsule. The head presents a small, rough depression posteroinferior to its center, called the fovea. The femoral neck is approximately 5 cm long and connects the head to the shaft at the neck-shaft angle, which measures around 127° on average. The neck-shaft angle facilitates movement at the hip joint, enabling the limb to swing clear of the pelvis. The neck also provides a lever for the action of the muscles acting about the hip joint, which are attached to the proximal femur. The neck is laterally rotated with respect to the shaft to around 10-15°, called the angle of anteversion, which has been found to vary between individuals and populations. The greater trochanter is a large quadrangular projection arising from the junction of the neck and shaft. The lesser trochanter is a conical posteromedial projection of the shaft at the postero-inferior aspect of its junction with the neck. The intertrochanteric line descends medially from the anterior aspect of the greater trochanter to a point on the lower border of the neck, anterior to the lesser trochanter. The intertrochanteric crest descends medially from the posterosuperior angle of the greater trochanter to the lesser trochanter [1].

It is often seen that major sources of evidence collected from crime scenes, burial grounds, sites of an explosion, and archaeological excavations are usually unknown fragmented skeletal remains. Stature estimation from such incomplete fragments of bone is a crucial step in determining the personal identity of the individual during such scientific investigations. The femur is also among the most frequent bones recovered from disaster sites [2, 3]. Also, it has been widely opined that the femur is one of the bones that shows the highest correlation with stature [2, 4, 5]. Linear regression formulae based on the length of extremity bones, particularly femurs, have been considered to be the best estimators of stature [6]. However, in situations when the entire bone is unavailable in intact condition, it becomes necessary to estimate the femoral length from available fragments. In such challenging situations, the proximal fragment of the femur with increased density of cortical bone and the better muscular cover is often found to be well preserved, making it available for recovery and forensic analysis [7]. This method which involves the initial estimation of the maximum length of the femur from its fragments followed by stature estimation, has been described as the indirect method of stature estimation [8]. Studies have shown significant positive correlations between measurements of the proximal femoral fragment and total femoral length [2, 3, 6]. This suggests that the measurement of proximal femoral parameters can be used to estimate total femoral length, which can then be used for stature estimation of the unidentified individual.

There is an increasing incidence of proximal femoral fractures nowadays due to low-velocity injuries in the elderly and high-velocity injuries from motor vehicle accidents in the younger population [9]. Optimal treatment of these fractures requires cephalomedullary nailing implants whose geometry must closely match the parameters of the proximal native femora [9, 10]. Hip joint arthroplasty is also a very commonly performed orthopedic surgical procedure these days due to the widespread prevalence of hip osteoarthritis and related hip ailments [11]. Successful treatment outcome in these scenarios requires stable anatomical fixation with proper alignment. The morphometric parameters of the femur have been found to be widely affected by race, sex, environmental factors, and lifestyle [11]. As most of the prostheses available in the market have been designed keeping Caucasian and Chinese parameters in mind, there is a mismatch between the dimensions of commercially available hip joint prostheses and with proximal femoral geometry of the Indian population [11, 12, 13]. Attempting joint fixation with such mismatched implants can result in complications, such as aseptic loosening, improper load distribution, discomfort, micromotion of the implanted stem, and stress shielding [13]. This further emphasizes the need to generate population-specific data that will be useful for designing adequately proportioned implants catering to the dimensions of the Indian population.

The primary objectives of the present study are (i) To measure various bony indices of the proximal femur, which will serve as a useful guide in designing customized implants and prostheses for the population of the Eastern Uttar Pradesh region; (ii) To observe significant correlation, if any, between the various measured bony parameters of proximal femur as well as to assess their correlations with total femoral length; and (iii)To derive a regression formula, specific to the population of eastern Uttar Pradesh region, for estimating total femoral length from the proximal fragment of the femur.

The findings of this study may provide further insight into resolving the existing lacuna of data in this subset of the Indian population.

## Materials and methods

Dry femora were procured from the Department of Forensic Medicine and Toxicology, Institute of Medical Sciences, Banaras Hindu University, Varanasi, Uttar Pradesh, India. These femora were collected from corpses brought to the department for medicolegal autopsy. Broken and damaged femora were excluded from the study. A total of 96 intact femora were included, out of which 51 were from the right side and 45 were from the left side. Measurements were made using a digital Vernier caliper (accuracy 0.001 mm). The femoral length was measured using an osteometric table (Bio Enterprises, Aligarh, India). The neck shaft angle was measured with an analog goniometer.

**Figure 1:**
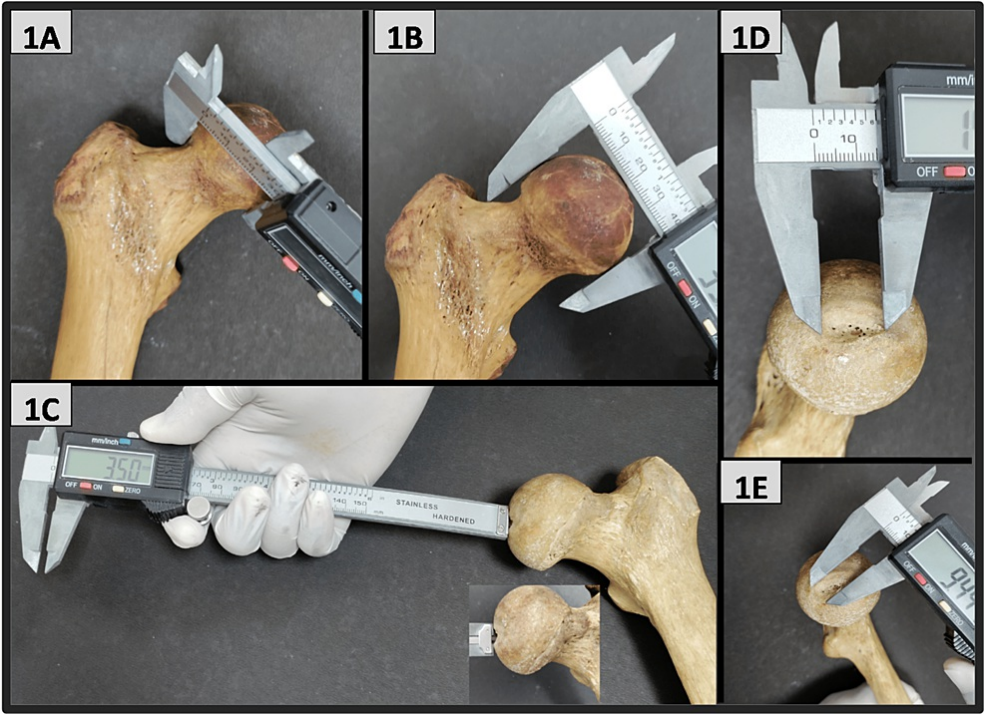
Measurements of different parameters of the proximal end of the femur by using a digital Vernier caliper. (1A) Femoral neck diameter (anatomical neck); (1B) Femoral head diameter; (1C) Foveal pit depth; (1D) Foveal longitudinal diameter; (1E) Foveal transverse diameter.

**Figure 2:**
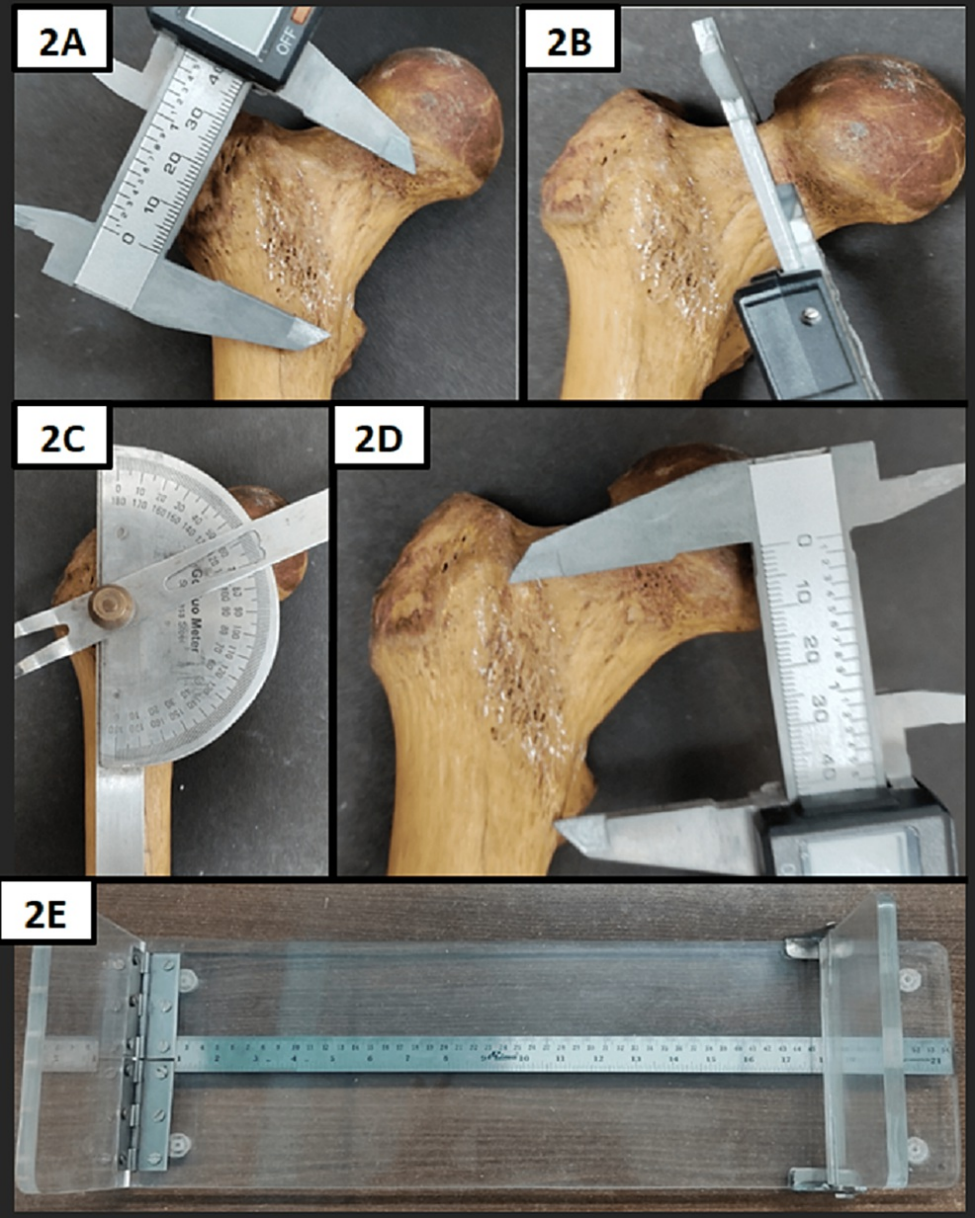
Measurements of different parameters of the proximal end of the femur by using Vernier caliper, Goniometer, and osteometric table. (2A) Femoral neck length; (2B) Femoral neck thickness; (2C) Femoral neck-shaft angle; (2D) Femoral intertrochentric line; (2E) Osteometric table.

The measurement of various parameters was taken as follows: (a) Head diameter was measured as the distance in a straight line between the upper end to the lower end of the femoral head in the craniocaudal axis (Figure 1B); (b) Foveal depth was measured as the maximum depth of the fovea capitis (Figure 1C); (c) Foveal transverse diameter and foveal longitudinal diameter are defined as the maximum extent of fovea capitis in transverse axis and vertical axis, respectively (Figure 1D-1E); (d) Neck diameter is the distance in a straight line from the upper end to the lower end of the anatomical neck of the femur in the craniocaudal direction (Figure 1A); (e) Neck length is the distance between the inferior region of the base of the femoral head and the lower end of the intertrochanteric line (Figure 2A); (f) Neck thickness is the thickness of the neck of the femur in anteroposterior axis (Figure 2B); (g) Neck shaft angle is defined as the angle between the axis of the neck and the axis of the shaft of the femur (Figure 2C); (h) Intertrochanteric line length is the total length of the intertrochanteric line (Figure 2D); and (i) Total femur length is the maximum length measured from the tip of the head of the femur to medial femoral condyle below (Figure 2E).

Data obtained was tabulated on a Microsoft Excel sheet and statistically analyzed using the IBM SPSS software version 25.

## Results

In the present study, the mean femoral head diameter was observed to be 41.59±3.25 mm, with a maximum of 47.32 mm and a minimum of 34.05 mm. Mean foveal depth was found to be 2.95±0.75 mm, maximum being 5.63 mm and minimum being 1.39 mm. Average foveal transverse and longitudinal diameters were observed to be 11.38±2.35 mm and 15.94±3.37 mm, respectively. The maximum and minimum foveal transverse diameters were 17.12 mm and 6.43 mm, respectively, while the foveal longitudinal diameter ranged from 6.73 mm to 24.59 mm. The mean neck diameter was 29.45±3.33 mm and ranged from 23.0 mm to 35.8 mm. Average neck length and neck thickness were observed to be 36.06±4.94 mm and 27.61±2.71 mm, respectively. The maximum neck length was 46.07 mm, and the minimum neck length was 25.59 mm. The maximum neck thickness was 33.38 mm, and the minimum neck thickness was 22.11 mm. Neck shaft angle was noted to range from 109° to 128°, with an average of 119.08°±5.18°. The mean length of the inter-trochanteric line was measured to be 41.92±3.9 mm, with a maximum of 51.53 mm and a minimum of 34.08 mm. The mean femoral length was 42.11±2.91 cm, with the longest femur measuring 54.2 cm and the shortest femur measuring 35.4 cm (Table 1).

**Table 1:** Descriptive statistical summaries of all the measurements of the femur.

Parameter	Mean ± SD	Minimum	Maximum
Head diameter (mm)	41.59 ± 3.25	34.05	47.32
Foveal depth (mm)	2.95 ± 0.75	1.39	5.63
Foveal transverse diameter (mm)	11.38 ± 2.35	6.43	17.12
Foveal longitudinal diameter (mm)	15.94 ± 3.37	6.73	24.59
Neck diameter (mm)	29.45 ± 3.33	23.00	35.80
Neck length (mm)	36.06 ± 4.94	25.59	46.07
Neck thickness (mm)	27.61 ± 2.71	22.11	33.38
Neck shaft angle	119.08 ± 5.18	109.00	128.00
Intertrochanteric line length (mm)	41.92 ± 3.90	34.08	51.53
Maximum femur length (in cm)	42.11 ± 2.91	35.40	54.20

The second objective of this study was to observe correlation among various parameters of the proximal femur, for which SPSS software (version 25) was used. The head diameter showed significant positive correlations with foveal longitudinal diameter, neck diameter, intertrochanteric line length, and femur length with a p-value <0.01. The head diameter was also significantly correlated with foveal depth, foveal transverse diameter, and neck thickness with a p-value <0.05. Foveal depth was significantly correlated with neck diameter with a p-value <0.01. Foveal depth was significantly correlated with head diameter, foveal longitudinal diameter, neck thickness, and femur length with a p-value <0.05. The foveal transverse diameter was significantly correlated with the foveal longitudinal diameter and intertrochanteric line length with a p-value <0.01. It also showed significant positive correlations with head and neck diameter at a p-value <0.05. The foveal longitudinal diameter was significantly correlated with the head and foveal transverse diameter with a p-value <0.01. It was also significantly correlated to foveal depth and intertrochanteric line length with a p-value <0.05. Neck diameter was significantly related to head diameter, foveal depth, neck thickness, and intertrochanteric line length with a p-value<0.01. It was significantly related to foveal transverse diameter and femur length with a p-value <0.05. Neck length was significantly related to neck thickness with a p-value <0.01. Neck thickness was significantly related to neck diameter with a p-value <0.01. It was significantly correlated to head diameter, foveal depth, neck length, intertrochanteric line length, and femur length with a p-value<0.05. Neck shaft angle was not significantly related to any other parameter. Intertrochanteric line length was significantly related to head diameter, foveal transverse diameter, and neck diameter with a p-value <0.01. It was significantly related to foveal longitudinal diameter and neck thickness with a p-value <0.05. Femur length was significantly related to head diameter with a p-value <0.01 and was significantly related to foveal depth, neck diameter, and neck thickness with a p-value <0.05 (Table 2).

**Table 2:** Pearson correlation analysis between the femur length and other femoral parameters. The highest positive correlation exists between the head diameter with neck diameter. Femur length is positively correlated to a varying degree with head diameter, foveal depth, neck diameter, and neck thickness, where a statistically significant correlation can be understood by the respective p-value <0.05. * Correlation is significant at the 0.05 level (2-tailed). ** Correlation is significant at the 0.01 level (2-tailed).

Parameters		Head diameter (mm)	Foveal depth (mm)	Foveal transverse diameter (mm)	Foveal longitudinal diameter (mm)	Neck diameter (mm)	Neck length (mm)	Neck thickness (mm)	Neck shaft angle	Intertrochanteric line length (mm)	Femur length (in cm)
Head diameter (mm)	r	1	0.213*	0.246*	0.324**	0.498**	0.046	0.250*	-0.137	0.346**	0.327**
	p		0.037	0.016	0.001	0	0.656	0.014	0.182	0.001	0.001
	Foveal depth (mm)	r	0.213*	1	0.178	0.241*	0.295**	0.093	0.258*	-0.099	0.11	0.239*
p		0.037		0.083	0.018	0.004	0.367	0.011	0.34	0.286	0.019
Foveal transverse diameter (mm)		r	0.246*	0.178	1	0.445**	0.216*	-0.036	0.114	-0.031	0.274**	0.089
	p	0.016	0.083		0	0.034	0.731	0.267	0.763	0.007	0.389
	Foveal longitudinal diameter (mm)	r	0.324**	0.241*	0.445**	1	0.028	0.042	0.145	-0.11	0.255*	-0.029
p		0.001	0.018	0		0.788	0.682	0.16	0.288	0.012	0.778
Neck diameter (mm)		r	0.498**	0.295**	0.216*	0.028	1	-0.014	0.292**	-0.009	0.360**	0.231*
	p	0	0.004	0.034	0.788		0.889	0.004	0.933	0	0.024
	Neck length (mm)	r	0.046	0.093	-0.036	0.042	-0.014	1	0.222*	0.027	0.132	0.169
p		0.656	0.367	0.731	0.682	0.889		0.03	0.793	0.2	0.101
Neck thickness (mm)		r	0.250*	0.258*	0.114	0.145	0.292**	0.222*	1	-0.084	0.241*	0.222*
	p	0.014	0.011	0.267	0.16	0.004	0.03		0.416	0.018	0.03
	Neck shaft angle	r	-0.137	-0.099	-0.031	-0.11	-0.009	0.027	-0.084	1	0.039	0.186
p		0.182	0.34	0.763	0.288	0.933	0.793	0.416		0.705	0.07
Intertrochanteric line length (mm)		r	0.346**	0.11	0.274**	0.255*	0.360**	0.132	0.241*	0.039	1	0.163
	p	0.001	0.286	0.007	0.012	0	0.2	0.018	0.705		0.112
	Maximum Femur length (in cm)	r	0.327**	0.239*	0.089	-0.029	0.231*	0.169	0.222*	0.186	0.163	1
p		0.001	0.019	0.389	0.778	0.024	0.101	0.03	0.07	0.112	

The third objective of the study was to formulate a regression formula to calculate femoral length using the bony parameters of the proximal femur. Table 3 demonstrates the model summary of multiple regression keeping the femur length as the dependent variable and other parameters as predictors. The R-value (multiple correlation coefficient) indicates the value of 0.507, which is a non-satisfactory level of prediction. The lower value of R-square and adjusted R-square also denotes a non-satisfactory model fit. The F-ratio value (3.301) indicates that the overall regression model is not a good fit for the data set as the significant value is 0.002 (greater than 0.0005) (Table 4). These findings suggest that the independent variables are statistically non-significant predictors of the dependent variable, i.e., femur length.

**Table 3:** The model summary of multiple regression keeping the femur length as the dependent variable and other parameters as predictors. The R-value (multiple correlation coefficient) indicates the value of 0.507, i.e., a non-satisfactory level of prediction. In contrast, the lower value of R-square and adjusted R-square denotes a non-satisfactory model fit. a. Predictors: (Constant), intertrochanteric line, Neck shaft angle, neck length, foveal depth, foveal transverse diameter, neck thickness, head diameter, foveal longitudinal diameter, and neck diameter. b. Dependent variable: femur length.

Model Summary^b^									
Model	R	R-Square	Adjusted R-Square	Std. Error of the Estimate	Change Statistics				
					R-Square Change	F Change	df1	df2	Sig. F Change
1	0.507a	0.257	0.179	2.63954	0.257	3.301	9	86	0.002

**Table 4:** The F-ratio value (3.301) indicates that the overall regression model is not a good fit for the dataset. The significant value, i.e., (0.002) greater than 0.0005, represents the independent variable statistically non-significant predictor of the dependent variable, i.e., femur length. a. Dependent variable: femur length b. Predictors: (Constant), intertrochanteric line, Neck shaft angle, neck length, foveal depth, foveal transverse diameter, neck thickness, head diameter, foveal longitudinal diameter, and neck diameter.

ANOVA^a^						
Model		Sum of Squares	df	Mean Square	F	Sig.
1	Regression	206.986	9	22.998	3.301	0.002^b^
	Residual	599.179	86	6.967		
	Total	806.165	95			

## Discussion

The incidence of total hip arthroplasties and hip revision surgeries has increased worldwide considerably over the past few years. It has been estimated, according to projection studies, that the demand for primary total hip arthroplasties is expected to grow by 174% by the year 2030 [14]. India has also witnessed an exponential rise in joint replacement surgeries over the last decade [14, 15]. Around 1000-2500 total hip arthroplasties have been performed on a yearly basis in India over the last decade, with increased utilization of uncemented total hip arthroplasty from 2006 to 2019 [15]. It has also been projected that many hip fractures occurring worldwide each year will reach 6.26 million by 2050 [16]. In this scenario of rising numbers of hip surgeries, the anthropometric measurements of the proximal femur can serve as a valuable tool for designing better fitting and well-adjusted femoral implants and prostheses to improve treatment outcomes.

A major component of successful total hip arthroplasty is the design of the femoral head prosthesis. Currently, though oversized heads are preferred due to decreased chances of dislocation, they can lead to numerous other complications like wear, imperfect biomechanics, and groin pain [17]. Hence, an accurate assessment of femoral head diameters with consideration of regional variations becomes indispensable for total hip replacement. In our study, the vertical head diameter was observed to be 41.59±3.25 mm. This tallies with studies done in southern India by Lingamdenne PE et al., Kamath SU et al., and Sengodan VC et al., where the average head diameter was 42.3±0.54 mm, 44.8±4.2 mm, and 42.6 mm, respectively [18-20]. Studies done in northern India by Verma M et al. and Siwach RC show an average head diameter of 42.32±4.11 mm and 43.95±3.06 mm, respectively [11, 12]. A study done in eastern India by Sengupta I et al. reported head diameter to be in the range of 38.56±2.5 mm to 38.07±3.43 mm [21]. In northeastern India, Saikia KC et al. found that the mean vertical head diameter ranged from 40.75 to 44.6 mm [22].

The fovea capitis is the site of attachment of an important ligament, ligamentum teres capitis, which contributes to joint stability. The blood vessels supplying the head of the femur and adjoining areas traverse through this ligament [23]. Therefore, the geometry of the foveal pit assumes great relevance, especially in avascular necrosis of the head of the femur. However, only scanty literature could be found regarding the study of fovea capitis. The foveal longitudinal diameter in our study was 15.94±3.37 mm, which is comparatively greater than the findings of Yarar B et al., who noted it to be 15.25±2.86 mm [24]. The same study observed a foveal transverse diameter of 12.00±2.17 mm, which is greater than our value of 11.38±2.35 mm [24]. The mean foveal pit depth in our study was 2.95±0.75 mm, which is greater than the value obtained by Yarar B et al., i.e., 2.67±1.13 mm [24].

The femoral neck assumes great importance in hip arthroplasties as it allows the femur to adjust to the new biomechanics of the prosthetic implant. In this regard, it is essential to remove only the pathological tissues while preserving as much osteo-architecture of the femoral neck as possible [25]. Also, a wide femoral neck is found to be associated with an increased risk of hip fractures in the elderly [26]. The average neck thickness in our study was 27.61±2.71 mm, which is less compared to Siwach RC’s findings in the North Indian population, where the average neck thickness was 31.87 mm [12]. However, Verma M et al. found a neck width of 24.01 ± 3.05 mm in the North Indian population [11].

The neck thickness values in our study are comparable with those found by Sengodan VC et al. in the South Indian population, i.e., 27.5 mm [20]. However, it is lesser when compared to findings by Sengupta I et al. in eastern India (28.84 mm-28.09 mm) [21]. The mean neck length in our study was calculated to be 36.06±4.94 mm, which is comparable with the maximum neck length of 37.23 ±4.65 mm found by Siwach RC, lesser than 44.75±8.097 mm found by Verma M et al., but greater than that of South Indian population by Isaac B et al., i.e., 28.4 mm [11,12,27]. The mean neck diameter in the present study measured 29.45±3.33 mm, which is consistent with the findings of Lingamdenne PE et al. in South India, who found it to be 29.6±0.26mm. However, it is lesser than data obtained from North India by Verma M et al., which measured 33.02±4.22 mm [11,18].

The neck shaft angle, which helps the acetabulum to align with the femoral head, is of great structural and diagnostic value in hip joint mechanics. The angle is a beneficial structural adaptation that increases hip rotation and helps the lower limb to swing away from the pelvis, increasing freedom of movement [27]. The average neck shaft angle in the present study was found to be 119.08°±5.18°. This is consistent with the findings of Lingamdenne PE et al., who measured it to be 119.44°±4.13° [18]. However, some of the other studies done by Verma M et al., Siwach RC, Kamath SU et al., and Sengodan VC et al. have reported higher values of the neck shaft angle ranging from 123.5°± 4.34° to 137.8°±6.9° [11,12,19,20]. The neck shaft angle varies with age and is influenced by several factors, including climate, occupation, race, ethnicity, and sedentary lifestyle [18, 20]. The neck shaft angle of the standard femoral prosthesis in arthroplasty is 131° [20], much greater than our study's mean value. This can cause increased difficulty while fixing the prosthesis during arthroplasty and can subsequently impair natural hip joint biomechanics during postoperative recovery.

The length of the femur contributes to 27% of the individual's stature [27]. The mean femur length in our study was found to be 42.11±2.91 cm. This is consistent with the findings of Kulkarni M et al. and Verma M et al., who observed it to be 41.95 ± 2.85 cm and 42.82±2.87 cm, respectively [9,11]. However, this value is lesser than that observed in studies by Chandran M et al. and Isaac B et al., who found the mean femoral length to be 44.9 cm and 43.4 ±2.7 cm, respectively [4,27].

Intertrochanteric fractures of the femur are a well-known occurrence, especially in the elderly population, and are mostly treated with surgical nail fixation [28]. Prasad R et al. have reported positive correlations between femoral length and intertrochanteric apical axis length, which was measured up to the midpoint of the intertrochanteric line [2]. However, we could not find any data in the scientific literature regarding the intertrochanteric line on the anterior aspect of the femur, which can again serve as a valuable predictor of femoral length in stature estimation. In our study, we measured the mean length of the intertrochanteric line to be 41.92±3.9 mm and showed positive correlations with the head diameter, neck diameter, and foveal transverse diameter of the femur.

The findings of our study show significant positive correlations between the various measured proximal morphometric parameters of the femur. The length of the femur showed a maximum positive correlation with the vertical head diameter, followed by the neck diameter, thickness, and foveal depth. This further establishes the correlation between proximal femoral parameters and femoral length, which can be a useful tool for stature estimation of skeletal remains. Our findings correlate with those of Prasad R et al., Chandran M and Kumar V, and Abledu JK et al., who observed significant positive correlations between femoral length and proximal femoral parameters in their studies [2,4,6]. Isaac B et al. found the neck shaft angle to be significantly and positively correlated with neck length, intertrochanteric apical axis length, and minimum femoral length but not with the vertical head diameter [27]. This differs from our study, where we did not find any correlations between the neck shaft angle and other parameters.

Limitations of this study

The present study was done on 96 femora, where multiple parameters were analyzed. Though significant positive correlations were obtained between some of these parameters and the length of the femur, a linear regression equation could not be formed between proximal femoral parameters and femoral length. In this regard, the sample size can be further expanded, and the data generated can be studied again for the application of linear regression formulae. Nevertheless, our study has found positive correlations between the proximal femoral fragment and femur length. Therefore, studies assessing the distal fragment of the femur with femoral length correlation may be planned in the future to give further insight.

## Conclusions

Many investigators have found femoral morphometry to vary with age, gender, race, ethnicity, and regional customs like sitting cross-legged or squatting. The skeletal parameters of the Indian population are well known to differ from other racial groups globally. The proximal femoral morphometry shows significant regional differences among Asian, Caucasian, and African populations. Though many authors have anthropometrically studied the proximal femoral fragment from different parts of the world, there are relatively fewer studies that have been done in India. India comprises a heterogeneous population of varying ethnic, genetic, and morphological subgroups. The findings of our study show that there are considerable variations in femoral parameters within different parts of India itself. Currently, no studies have assessed femoral anthropometry from the eastern Uttar Pradesh population. Our study attempts to address this lacuna of data and is the first to present a detailed analysis of multiple parameters of the proximal femoral fragment in this population. This may give valuable insight into a better-customized implant and prosthesis design.
